# Three-Year Outcomes of Cross-Linking PLUS (Combined Cross-Linking with Femtosecond Laser Intracorneal Ring Segments Implantation) for Management of Keratoconus

**DOI:** 10.1155/2018/6907573

**Published:** 2018-01-17

**Authors:** Mohammed Iqbal Hafez Saleem, Hosam A. Ibrahim Elzembely, Mortada Ahmed AboZaid, Mohammed Elagouz, Ahmed Mohamed Saeed, Osama Ali Mohammed, Ahmed Gad Kamel

**Affiliations:** ^1^Faculty of Medicine, Sohag University, Sohag, Egypt; ^2^Faculty of Medicine, Minia University, Minia, Egypt; ^3^Faculty of Medicine, Banha University, Banha, Egypt

## Abstract

**Purpose:**

To analyze the results of three-year outcomes of combined epithelium-on cross-linking with femtosecond laser ICRS (cross-linking PLUS) for keratoconus management.

**Design:**

A retrospective multicenter clinical study.

**Methods:**

43 eyes of 38 patients were subjected to preoperative and postoperative UCVA, BCVA, refraction, Pentacam pachymetry, and keratometry examinations at 3-, 6-, 12-, 24-, and 36-month follow-up period.

**Results:**

The preoperative and postoperative mean UCVA was 1.30 ± 0.48 (logMAR ± SD) and 0.82 ± 0.22 respectively. The preoperative and postoperative mean BCVA was 0.90 ± 0.40 and 0.60 ± 0.30, respectively. The preoperative and postoperative mean K average was 50.63 ± 0.87 (D ± SD) and 45.56 ± 0.98, respectively. The preoperative and postoperative mean pachymetry was 471 ± 92.36 (*μ*m ± SD) and 423 ± 39.58, respectively. The preoperative and postoperative mean astigmatism was 7.55 ± 1.75 and 3.39 ± 1.26, respectively. One eye showed ICRS edge exposure while 6 eyes showed progression of keratoconus.

**Conclusion:**

CXL PLUS was proved to be a successful procedure to halt progression (mainly by CXL) and to correct the refractive status of the keratoconic eye (mainly by ICRS). CXL PLUS performed a synergistic action correcting and maintaining the correction of both myopic and astigmatic components of keratoconus.

## 1. Introduction

The most important clinical signs that characterize keratoconus (KC) are the irregular-marked astigmatism with progressive apical protrusion due to progressive thinning of the corneal stroma [1–3].

Many authors prefer to classify the severity of keratoconus according to the mean keratometry readings. The Amsler-Krumeich classification graded keratoconus into 4 grades depending on the main K readings. Grade 1 included mean central K readings < 48 diopters, grade 2 included mean central K readings ≥ 48–< 54 diopters, grade 3 included mean central K readings ≥ 54–< 55 diopters while grade 4 included mean central K readings ≥ 55 diopters [4].

One of the most trustable treatment modalities in keratoconus management is the implantation of intracorneal rings (ICRS). Actually, ICRS gain more ground and popularity every day. The long-term stability results of ICRS together with the use of the femtosecond laser devices actually encouraged surgeons to implant ICRS simply and safely. Furthermore, they had a good impact on the patient satisfaction after surgery [3–7].

ICRS are one of the most commonly used rings all over the world. The use of one or two segments in segmented ICRS or complete rings depends on the type of the keratoconus cone, corneal topography patterns, specially designed nomograms, and the surgeons' experiences. The main idea of ICRS implantation is to flatten the cornea and reduce anterior corneal surface irregularity, but they actually have no role in halting the progression of keratoconus [5–8].

Corneal collagen cross-linking (CXL) is the actual and main treatment to keratoconus and has the advantage of halting the progression of the pathology of the disease. The progression of keratoconus can be defined by continues change in 2 or more of special parameters. These special parameters included steepening of the posterior K readings, steepening of the anterior K readings, and thinning of the central pachymetry readings and high back surface elevations [5]. Cross-linking PLUS (CXL PLUS) is defined as the simultaneous combination of CXL and a refractive procedure to flatten the cornea and improve vision as ICRS implantation.[6]

Recently, CXL PLUS has become more popular among surgeons as it has the advantages of both halting KC progression and improving the visual outcome. CXL is the main mandatory procedure that stabilizes the cornea and halts the disease progression, hence the name CXL PLUS as plus means adjuvant refractive procedure to improve vision which could be ICRS implantation, topography-guided PRK, toric implantable collamer lens (TICL), phakic intraocular lens (IOL), or even refractive lens surgery (clear lens extraction) with toric IOL implantation. Patient selection for the suitable refractive procedure is essential as not all previously mentioned refractive procedures are suitable for all patients as every keratoconic eye has its suitable refractive procedure. CXL PLUS is the most beneficial modality of treatment as the use of the combination therapy depends on many factors mainly the degree of myopia and astigmatism and the corneal pachymetry and keratometry readings [9].

## 2. Patients and Methods

This study was designed as a retrospective multicenter study that aimed to analyze the three-year outcomes of CXL PLUS (combined CXL and Keraring implantation with the use of femtosecond laser) concerning its efficacy and safety.

This study was a multicenter study that was carried on 3 universities including Sohag University Hospital, Banha University Hospital, and Minia University Hospital in Egypt. This study had the approval of the ethical committee in Sohag University Hospital.

Only eyes that completed 3-years of follow-up are included in this study as all eyes were performed and followed up during the period from January 2014 to May 2017. All follow-up results of the medical records were obtained retrospectively for analysis of the patients' data.

43 eyes of 38 patients diagnosed with keratoconus were operated with CXL PLUS that included simultaneous combination of accelerated epithelium-on CXL and femtosecond laser Keraring implantation at the same session.

The 43 eyes were subjected to preoperative and postoperative UCVA, BCVA, manifest refraction, and Pentacam pachymetry and keratometry at 3-, 6-, 12-, 24-, and 36-month follow-up period.

The devices that were used in this study included CSO SIRIUS Topographer (CSO, Firenze, Italy), Advanced Femtosecond Laser (iFS; Abbott, USA), and the KXL System (Avedro Inc., MA, USA).

### 2.1. Surgical Procedure of CXL PLUS

CXL PLUS included combined femtosecond laser Keraring (Mediphacos Inc., Belo Horizonte, Brazil) implantation as the first step in surgery followed immediately by accelerated epithelium-on CXL as the second step in the same session. The data that was introduced into the device included the parameters of the corneal tunnel creation with an outer diameter of 5.9 mm and an inner diameter of 5 mm; the incision site was at the steepest corneal meridian and the tunnel was created at the depth of 75% of the corneal thickness at the thinnest location.

**Figure 1:**
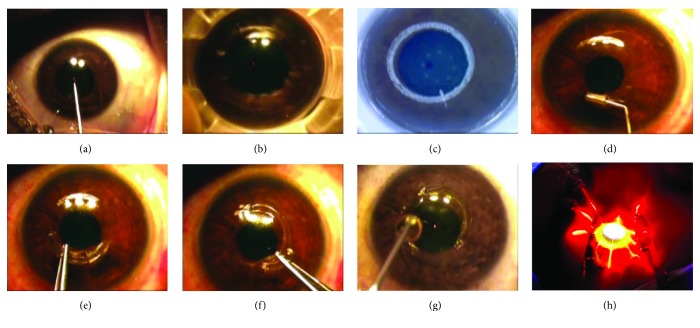
CXL PLUS in the right eye; (a) marking the corneal center when the patient is fixating at flashing light, (b) suction ring application onto the cornea, (c) corneal tunnel creation using the femtosecond laser, (d) checking the patency of the tunnel by passing a spatula through the limbs of the tunnel, (e) implantation of the temporal Keraring segment, (f) implantation of the nasal Keraring segment, (g) riboflavin dropping onto the cornea, and (h) accelerated epithelium-on CXL.

The specification of the Keraring used in this study was the SI-5 design (5.0 mm optical zone) with triangular design in cross-section, made of polymethyl methacrylate (PMMA). The use of one or two Keraring segments depended upon the Keraring nomogram. Different parameters affected the choice of one or two segmented rings mainly the type and the site of the keratoconus cone, the mean K readings, the central pachymetry readings, the corneal thickness at the thinnest location, and the refractive status of the eye. This study included only a sample of keratoconic eyes that completed the 3-year follow-up period and were treated relying upon the designed Keraring nomogram. Although all eyes included in this study were treated with an implantation of 2 Keraring segments, yet it should be clear that this was not the concept to be generalized as a rule for all keratoconic eyes. Moreover, the characteristics of these eyes included in this study required implantation of 2 Keraring segments in each eye using the Mediphacos Nomogram for Keraring calculation guidelines 2009 version 5.2.  Figure 1 shows an example of CXL PLUS in the right eye.

The first step started with marking the corneal center when the patient is fixating at the flashing light (Figure 1(a)), then followed by application of the suction ring onto the cornea with great care of corneal centralization within the suction ring (Figure 1(b)). The corneal tunnel was created using the femtosecond laser with a power of 5 mJ (Figure 1(c)). Passing a spatula was performed through the limbs of the tunnel to check its patency (Figure 1(d)). The temporal and nasal Keraring segments were implanted (Figures 1(e) and 1(f)).

The second step was to perform the epithelium-on CXL using Avedro Nomogram with Avedro KXL CXL system. Accelerated epithelium-on CXL included installation of riboflavin (ParaCel) on the cornea every 90 seconds which is repeated 3 times along duration of four and half minutes. The next step was to install another type of riboflavin (Vibex Xtra) that was also performed every 90 seconds which is repeated 4 times along a duration of six minutes. The total soaking time was 11 minutes. Accelerated epithelium-on CXL was performed by using the pulsed UV mode (1 second on 1 second off) with UV time 2 minutes and 40 seconds while the total treatment time was 5 minutes and 20 seconds at a power of 45 mW/cm^2^ to deliver a total energy of 7.2 J/cm^2^. Application of bandage contact lens onto the cornea and installation of antibiotic eye drops (moxifloxacin 0.5%) into the eyes were performed at the end of the surgery. Dropping of riboflavin onto the cornea (Figure 1(g)). CXL using the accelerated epithelium-on procedure (Figure 1(h)).

### 2.2. Postoperative Treatment

All patients received the same postoperative treatment which lasted for three weeks. The postoperative regimen included topical therapy in the form of corticosteroid eye drops (prednisolone acetate 1%) in addition to antibiotic eye drops (moxifloxacin 0.5%) and artificial tears (hypotonic sodium hyaluronate 0.15%). All eye drops were prescribed for all patients on 2 hourly bases on the first postoperative day then 4 times daily in the first week. The frequency of medication was tapered to be 3 times daily in the second week and twice daily in the third week. Bandage contact lens was removed at the first postoperative day.

### 2.3. Statistical Analysis

Analysis of the data was performed using the statistical package for social sciences software (SPSS version 22 for Windows). The description of the quantitative data was by using the median, mean and standard deviation. The presentation of the qualitative data was in the form of number and percentage. Paired sample *t*-test was used for normally distributed data while Wilcoxon test was used for nonnormally distributed data. The postoperative results were considered to be significant at the 5% level.

## 3. Results

This study included 43 eyes of 38 patients. 23 patients were females (60.5%) while 15 patients were males (39.5%). All patients fall in the age group from 14 to 25 years with a mean age of 19.58 ± 4.05 (mean ± SD).  Table 1 shows the characteristics of the study patients.

Two segments of Kerarings were implanted in all 43 keratoconic eyes in this study. Keraring model SI-5 (5 mm optical zone) segments were used in this study.

All preoperative and postoperative data collected from the patient's files over a follow-up period of 36 months were summarized in Table 2.

This study showed the great influence of the CXL PLUS in reducing the mean K average that was reduced from 50.63 ± 0.87 diopters (mean ± SD) preoperatively to 45.56 ± 0.98 postoperatively (*P* value < 0.05). This amazing reduction in the mean K average to approximately 5 D can be attributed to the action of both Keraring implantation and CXL.

Furthermore, these 5 diopters reduction in the mean postoperative K average resulted from the combination of correction in both mean postoperative myopic and astigmatic correction. As the results in this study indicated the correction of postoperative myopic component up to 1 D approximately while the marked correction was shown in the postoperative astigmatic component up to a level of 4 D approximately.

These good results were reflected on the patient both UCVA and BCVA postoperatively. This study showed improvement of the preoperative mean UCVA from 1.30 ± 0.48 (logMAR ± SD) to a postoperative mean UCVA 0.82 ± 0.22 (*P* value < 0.05). Moreover, the preoperative mean BCVA 0.90 ± 0.40 improved to be 0.60 ± 0.30 postoperatively (*P* value < 0.05). All patients showed improvement two lines or more during the visual chart testing in both UCVA and BCVA.

This study also showed the reduction of the preoperative mean corneal thickness at the thinnest location 471 ± 92.36 *μ*m (mean ± SD) to a postoperative level of 423 ± 39.85 (*P* value < 0.05). The reduction in the postoperative mean corneal thickness was mostly due to 2 main reasons. The first reason was due to the efficacy of the epithelium-on CXL, and reduction in the postoperative mean corneal thickness was an indication of its effectiveness. The second reason was that in 6 eyes further keratoconus progression was recorded manifested by an increase in the postoperative K readings and reduction in the corneal thickness.

Table 3 shows a comparison between the preoperative and postoperative data of one female patient after 3 years of CXL PLUS.

### 3.1. Complications

Complications were recorded in 7 eyes (16.3%) in this study. One eye was complicated by exposure of the edge of the Keraring segment within the first postoperative month so that the ring was removed and the procedure was repeated 3 months later with success.

Another complication that was recorded in this study was the progression of keratoconus and deterioration of the condition in 6 eyes so that the cross-linking procedure was repeated in all 6 eyes using the standard conventional 30 minutes epithelium-off CXL.

Six eyes (14% of eyes) showed progression of keratoconus, the CXL was repeated in these 6 eyes using the standard conventional 30 minutes CXL procedure (The Dresden Protocol), and the cornea was exposed to UV irradiation using Opto XLink-Corneal Crosslinking System (Opto Global Pty Ltd., Adelaide, Australia). The UV irradiation was performed at a power of 1.50 mW, an intensity of 2.984 mW/cm^3^, and a dose of 5.371 J/cm^3^ for 30 minutes. 8 mm deepithelized. corneal zone was performed for all 6 eyes followed by dropping of riboflavin VibeX Rapid™ (Avedro Inc., MA, USA) every 3 minutes for 30 minutes then corneal irradiation with UVA for 30 minutes with continuation of riboflavin dropping during corneal irradiation.

The eyes were followed during the period of the study to complete the 3-year follow-up and surprisingly corneal flattening occurred with improvement of the K readings and further thinning of the corneal thickness. So that the accelerated epithelium-on CXL was effective in 37 eyes to halt the progression of keratoconus (86% of eyes) while it failed to stop the deterioration of 6 keratoconic eyes (14% of eyes) that was treated with standard conventional 30 minutes epithelium-off CXL.

According to the postoperative complications, the 43 keratoconic eyes included in this study were divided into 3 postoperative groups according to the results:

Group A included 36 eyes (83.7% of eyes) that were subjected to CXL PLUS using simultaneous epithelium-on CXL and 2 Keraring segments implantation and showed good improvement with stability of the results along the 3-year follow-up period.

Group B included 6 eyes (14% of eyes) that were subjected to CXL PLUS using simultaneous epithelium-on CXL and 2 Keraring segments implantation but showed further deterioration within the first 3 months postoperatively so that these 6 eyes were subjected to additional conventional standard epithelium-off CXL procedure which managed to stop further KC deterioration and showed good stability and further improvement along the 3-year follow-up period.  Table 4 shows the detailed refractive data of these 6 eyes along the whole study period.

Group C included 1 eyes (2.3%) was complicated by Keraring exposure during the first postoperative month. The exposed segment was removed together with the second segment in the same session. Three months later, another 2 Keraring segments were implanted. The eye was followed up for 3 years and showed good improvement and stability of the condition.  Table 5 shows the detailed refractive data of this eye along the whole study period.

Table 6 shows the summary of postoperative complications.

## 4. Discussion

The reliability of the results of this study arises from the long-term follow-up period (3 years).

CXL PLUS was proved to be effective and relatively safe in this study. The use of Kerarings for mechanical corneal flattening was effective in reducing the postoperative K readings.

Analysis of the preoperative and postoperative data of the patients at the 36th postoperative follow-up months was performed. The results of this analysis together with collecting the missing tiny points helped this study to make a clear impression upon the CXL PLUS. This study showed clearly that Keraring was very effective in flattening the steep corneal meridians thus reducing markedly the astigmatic component of KC and help correcting the anterior corneal surface irregularities. Furthermore, the use of Kerarings improved the postoperative astigmatism up to 5 D or even more in few cases. The procedure of the accelerated epithelium-on was originally introduced to stop deterioration of the disease and was not expected to improve vision or even flattening the cornea.

The discrepancy between the two CXL procedures (epithelium-on and epithelium-off) showed the importance of reevaluating the two procedures as it seemed that the conventional 30 minutes epithelium-off CXL was more effective in halting the KC progression and flattening of the corneal surface thus sharing in correcting the myopic component of KC and reducing the anterior irregularities. On the contrary, the accelerated epithelium-on CXL was relatively effective in halting the KC progression in most cases while in some cases it was not actually effective to stop the disease progression.

Many authors had proved in their studies the effectiveness of Keraring implantation regarding better visual acuity and decreasing the spherical equivalent and keratometry readings. One of them was Gharaibeh et al. [8] which reported the importance of ICRS in delaying or preventing keratoplasty. Other studies as Coskunseven et al. [7] reported the accuracy and safety of Keraring implantation using the femtosecond laser and stressed on its success in improving both UCVA and BCVA. Furthermore, their study reported the easy learning curve for the beginner surgeons with high patient satisfaction and comfortability. Their results were similar to the results in this study as the femtosecond laser (iFS, Abbott) was used for tunnel creation that facilitates Keraring implantation with almost no intraoperative patient complains or discomfort.

This study did not include the use of Keraring 355° meanwhile many other studies reported its use in treating KC. Jadidi et al. [3] reported the use of Keraring 355° with reduced risk of intraoperative complications and concluded the efficacy and safety of this type of Keraring.

One of the most interesting studies were Kubaloglu et al. [10, 11] which proved the success of Keraring implantation in reducing the spherical equivalent and decreasing the Kmax to a level of 4 D after a follow-up period of 6 months. This study coincided with Kubaloglu et al. [10, 11], yet our study showed a reduction of the astigmatic component of KC to a level of 5 D or even more at a longer follow-up period of 36 months. One major difference between the two studies was that this study used combined Keraring implantation with epithelium-on CXL (CXL PLUS) while their study included only implantation of Kerarings.

In Alexandria, Ibrahim and colleagues [12] had similar results in their study as they reported the marvelous results of combined epithelium-on CXL and Keraring implantation using femtosecond laser as an effective procedure that improved vision and decreased the anterior corneal surface irregularity. Although their results were similar to this study however, there were two main differences between both studies. The first difference was that they proved the efficacy of the procedure in lowering the main spherical equivalent refractions, while in this study it was more clear regarding the efficacy of the procedure on both myopic and astigmatic components of KC as it proved that this procedure was much more effective in correcting the astigmatic component than the myopic component of KC instead of the use of the spherical equivalent refraction terminology. The second difference was noted in their study as they found no significant reduction in the corneal thickness postoperatively while this study proved that there was a significant reduction in the postoperative corneal thickness. Furthermore, in this study, the follow-up period was 3 years while in their study was 6 months.

Shawky et al. [13] reported interesting results in their research as they studied the effect of performing CXL few months after Keraring implantation. They noted the good effect of CXL in improving both spherical and cylindrical elements of KC. Furthermore, they reported lowering the corneal astigmatism up to 2.1 D and up to 3.5 D in the main keratometric reading. Their results coincided with the results of this study; however, this study showed a greater reduction in the postoperative corneal astigmatism reaching up to 4 D or more and a further greater reduction in the mean K average reading up to 4 D or more. Moreover, they stated that CXL had a stabilizing enhancement after Keraring implantation but this study of CXL PLUS was performed using CXL and Keraring implantation in the same session as CXL is almost mandatory for all keratoconic eyes.

Other authors reported the results of CXL PLUS using different techniques by combining CXL and topography-guided photorefractive keratectomy (PRK) whether in the same session or sequential. Bor'i [14] reported that there was no significant difference between simultaneous or sequential CXL PLUS using combined CXL and PRK. In another interesting study, Abou Samra et al. [15] compared also the results of two study groups regarding simultaneous versus sequential wavefront-guided (WFG) PRK. They followed up patients for 6 months postoperatively and finally concluded that there were no significant differences between both groups.

Moreover, El-Raggal [16] compared CXL PLUS in two groups. The first group had epithelium-on CXL and Keraring implantation in the same session while the second group had Keraring implantation first and then followed by epithelium-on CXL later. He reported close results in both groups. However, his study proved the superiority of CXL PLUS when CXL was combined with Keraring implantation in the same session. He explained this superiority by assuming that riboflavin reaches corneal stroma more effectively through passing in the channels created for Keraring implantation. His results are in one line of the results of this study regarding safety and efficacy of CXL PLUS.

In another comparative study, Iqbal [17] compared two techniques; the first technique was combined CXL with myoring implantation while the second technique was combined CXL with Keraring implantation. He concluded that Keraring is more successful in reducing the astigmatism than myopia in keratoconus. On the contrary, his study reported that myoring is more successful in reducing myopia than astigmatism in keratoconus. The results of his study coincided partially with the results of this study regarding combined CXL with Keraring implantation (CXL PLUS).

Furthermore, in a different comparative study to the same author, Iqbal [18] compared epithelium-off CXL with epithelium-on CXL without ICRS implantation. His study reported that epithelium-off CXL is superior to epithelium-on CXL regarding both halting KC progression and inducing corneal flattening thus improving both visual acuity and the myopic component of KC. This study coincided partially with his study regarding the effectiveness of the conventional epithelium-off CXL and its superiority upon accelerated epithelium-on CXL which had failed to halt KC progression in 14% of the study eyes and the procedure was repeated in these eyes using the conventional epithelium-off CXL that managed to halt KC progression along a 36-month follow-up of this study.

Moreover, many authors proved in their studies that epithelium-off CXL was more effective that transepithelial CXL. Kocaka [19] reported that epithelium-off CXL was more effective than epithelium-on CXL in halting KC progression and improving the refractive status in the eye. Furthermore, Soeters et al. [20] concluded that transepithelial CXL was a safe procedure; however, KC progression was recorded in 23% of cases so that they recommended continuing using epithelium-off CXL and not to shift to epithelium-on CXL. The results of their study are close to but slightly higher than the results in this study which recorded KC progression in 14% of eyes after accelerated epithelium-on CXL.

## 5. Conclusion

CXL PLUS proved to be a successful procedure to halt progression (mainly by CXL) and to correct the refractive status of the keratoconic eye (mainly by ICRS). CXL PLUS performed a synergistic action correcting and maintaining the correction of both myopic and astigmatic components of keratoconus. This study proved that Keraring implantation is more effective in lowering the astigmatic component of KC than the myopic component. This study recorded KC progression in 14% of cases following CXL PLUS with the accelerated epithelium-on CXL so that it is recommended to perform new prospective comparative studies in the future comparing both epithelium-on CXL and epithelium-off CXL procedures in CXL PLUS for the treatment of keratoconus.

## Figures and Tables

**Table 1:** Characteristics of the study patients.

Item	Number	Percent
Total number of study eyes	43	
Total number of study patients	38	
Age		
Range	14–25 years	
Mean ± SD	19.58 ± 4.05	
Gender		
Male	15 patients	39.5%
Female	23 patients	60.5%
Laterality		
OD	18 eyes	41.9%
OS	25 eyes	58.1%

**Table 2:** CXL PLUS preoperative and postoperative data summary at the end of the 3-year follow-up.

Parameters	Preoperative data (mean ± SD)	Postoperative 36th month data (mean ± SD)	*P* value
Mean K reading average (D)	50.63 ± 0.87	45.56 ± 0.98	<0.05
Mean K1 reading	46.94 ± 0.26	46.45 ± 0.98	<0.05
Mean K2 reading	54.38 ± 1.17	47.39 ± 0.62	<0.05
Mean corneal thickness at thinnest location (μm)	471 ± 92.36	423 ± 39.85	<0.05
Mean postoperative myopic correction (D)	—	0.97 ± 0.28	
Mean astigmatism (D)	7.55 ± 1.75	3.39 ± 1.26	<0.05
Mean postoperative astigmatic correction (D)	—	3.8 ± 1.67	
Mean UCVA (logMAR)	1.30 ± 0.48	0.82 ± 0.22	<0.05
Mean BCVA (logMAR)	0.90 ± 0.40	0.60 ± 0.30	<0.05

**Table 3:** A comparison between the preoperative and postoperative data of one female patient after 3 years of CXL PLUS.

Variant	Preoperative			Postoperative (36^th^ month)		
Corneal topography (tangential anterior)	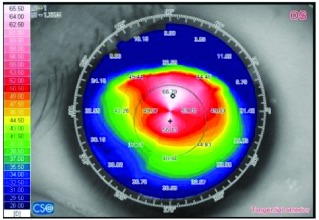			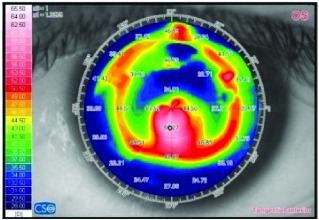		
K average (D)		50.39			47.075	
K readings (D)	47.52		53.26	46.08		48.07
Subjective refraction	−2.57		−7.00 @ 96	−0.50		−0.75 @ 20
Corneal thickness at the thinnest location (μm)		402			387	
Myopic component (D)		2.75			2.00	
Astigmatic component (D)		5.74			1.00	
Myopic correction		—			0.75	
Astigmatic correction		—			4.74	
UCVA (logMAR)		1.30			0.40	
BCVA (logMAR)		0.52			0.22	

**Table 4:** The summary of the refractive data of the six complicated eyes (group B).

Parameters	Preoperative data (mean ± SD)	3 months following CXL PLUS (mean ± SD)	36 months following epithelium-off CXL (mean ± SD)
Mean K reading average (D)	49.52 ± 0.93	50.18 ± 0.59	48.06 ± 0.42
Mean corneal thickness at thinnest location, (*μ*m)	453 ± 37.81	429 ± 31.05	399 ± 53.89
Mean postoperative myopic correction (D)	—	—	1.02 ± 0.47
Mean astigmatism (D)	5.96 ± 1.58	6.46 ± 0.87	4.62 ± 1.14
Mean postoperative astigmatic correction (D)	—	—	1.76 ± 0.25
Mean UCVA (logMAR)	1.12 ± 0.60	1.30 ± 0.52	0.90 ± 0.28
Mean BCVA (logMAR)	0.82 ± 0.30	1.00 ± 0.40	0.70 ± 0.25

**Table 5:** The summary of the refractive data of the 7^th^ complicated eye (group C).

Parameters	Preoperative data	Following removal of the 2 Keraring segments	36 months following reimplantation of 2 new Keraring segments
Mean K reading average (D)	51.37	52.04	48.06
Mean corneal thickness at thinnest location, (*μ*m)	428	426	421
Mean postoperative myopic correction (D)	—	—	1.25
Mean astigmatism (D)	5.75	5.50	1.50
Mean postoperative astigmatic correction (D)	—	—	1.00
Mean UCVA (logMAR)	1.30	1.30	0.70
Mean BCVA (logMAR)	0.60	0.60	0.30

**Table 6:** The summary of complications.

Complication	Number of eyes	Percent	Management of the complications	Follow-up results along the 36 months
Postoperative KC progression	6	14%	Standard 30 minutes epithelium-off CXL	Improvement with halting KC progression
Exposure of the Keraring edge	1	2.3%	Explanation of the 2 Keraring segments followed by reimplantation of the Keraring segments 3 months later	Improvement with stability of the segments
